# The future is sweet: exploiting sugar allocation and use for cotton breeding

**DOI:** 10.1093/plcell/koag085

**Published:** 2026-03-20

**Authors:** Pablo González-Suárez

**Affiliations:** Assistant Features Editor, The Plant Cell, American Society of Plant Biologists; Department of Developmental Genetics, Centre for Plant Molecular Biology (ZMBP), Eberhard Karls University, Tuebingen D-72076, Germany

It is only natural for parents to worry about their children and make sure that they grow strong and healthy, ready to tackle any challenges they encounter. In their own way, plants also have strategies to ensure that their offspring grow vigorously. In the process, they must carefully allocate their resources among their successors, providing enough material for seeds to form and mature. Over years of evolution, this has led to a series of source-sink relationships, whereby nutrients are transported from the mother plant's tissues toward developing fruits (Fig. 1) (Braun et al. 2014). For crops, however, the interests of the mother plant do not always align with those of the agronomist. Cotton (*Gossypium hirsutum*) is grown worldwide for its oil- and protein-rich seeds and, importantly, its fibers. Historically, breeders have favored varieties with higher fiber yield and quality. Directing more resources to fibers sometimes comes at the cost of fewer nutrients allocated to seeds and thus a loss in seed yield.

**Figure 1:**
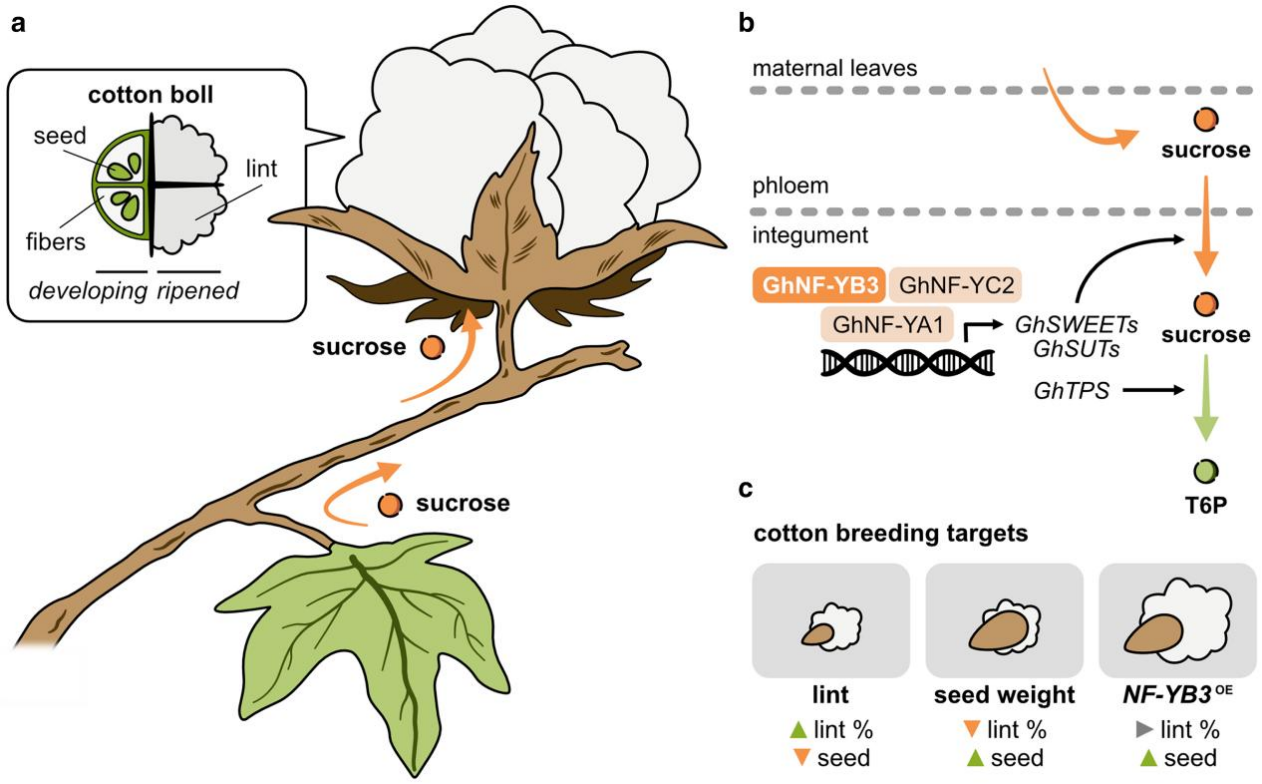
GhNF-YB3 controls sugar transport and use in the developing ovules of cotton. **a)** Diagram highlighting sucrose transport from source maternal leaves to cotton bolls. **b)** Model proposed by Wu et al. (2026) where GhNF-YB3 facilitates expression of sucrose transporters and T6P biosynthetic enzymes. **c)** Schematic showcasing potential targets for cotton breeding and how manipulations of GhNF-YB3 can be used to maximize seed yield without impact on lint production. SWEET (Sugars Will Eventually be Exported Transporter), SUT (Sucrose Transporter), TPS (Trehalose 6-Phosphate Synthase), T6P (Trehalose 6-Phosphate), OE (overexpression). Figure credit: P. González-Suárez.

Among the nutrients transported from maternal leaves to nurture the next generation of seeds, sucrose stands out as the main carbon source. After reaching its final destination in the ovule, sucrose can be metabolized to produce trehalose-6-phosphate (T6P), which serves as the key signal that promotes growth of the developing seeds. Though numerous studies have highlighted the importance of T6P signaling for yield (Paul et al. 2018), a greater understanding of its molecular underpinnings in species of agronomical interest would directly benefit crop improvement efforts.

The recent work by **Wu and coauthors (Wu et al. 2026)** opens an interesting avenue for cotton breeding. Equipped with a combination of biochemical and genetic tools, they set out to characterize a locus previously associated with seed yield variation among cotton cultivars (Zhao et al. 2023). The gene, now named *GhNF*-*YB3*, encodes a nuclear factor Y (NF-Y) subunit that is specifically expressed in the developing ovule. The authors demonstrate that, similarly to other NF-Ys, GhNF-YB3 forms a heterotrimer sequentially by first dimerizing with GhNF-YC2 and later with GhNF-YA1, both in vitro and in planta. The final heterotrimeric complex binds DNA and promotes seed development in at least 2 ways. In the vasculature and integument, it induces the expression of transporters that unload sucrose into and within the ovule. Further, the NF-Y complex activates T6P biosynthetic enzymes that further increase sugar allocation and use in the cotton boll (Fig. 1).

By regulating sucrose partitioning from maternal tissues to ovules, GhNF-YB3 has the potential to boost seed growth and nutritional quality. Accordingly, overexpressing and knockout lines developed by the authors show increased and decreased seed weight, respectively. Excitingly, this seems to come at no agronomic cost. Seeds from plants overexpressing GhNF-YB3 are bigger and contain increased protein levels without a reduction in fiber yield or seed numbers, highlighting GhNF-YB3 as a promising target for cotton breeding.

Having established the identity of *GhNF-YB3*, the authors ask whether differences in this locus among cotton cultivars may affect the gene's function. Inspecting genomic data from 200+ upland cotton accessions, they identify 4 polymorphisms in the promoter and 3′UTR, which together define 2 major haplotypes. Interestingly, these variants seem to impact the expression of the gene based on transient expression assays. The rarer haplotype, present in ∼20% of accessions, significantly enhances *GhNF-YB3* expression, potentially leading to higher sugar allocation and use in the ovule. In accordance, accessions carrying this haplotype show greater seed yield. Surprisingly, genomic analyses performed by the authors suggest that *GhNF-YB3* has not been under strong selection during domestication and that the higher-yielding haplotype is yet to be incorporated by cotton farmers.

In light of the ever-growing world population, it is imperative to make informed efforts to improve the efficiency of agricultural systems. As the world's main fiber crop, cotton production is no exception. Source-sink relationships between maternal tissues and the developing fruits remain a promising aspect to exploit. Through their work, Wu et al. (2026) take a step in the right direction, one that may be directly actionable to breeders and farmers worldwide.

## Recent related articles in *The Plant Cell*

- Liu et al. (2025) demonstrated a key role for alkaline α-galactosidase 2 in mediating raffinose metabolism and sugar allocation to developing seeds of cucumber.
- Wei et al. (2025) showed that natural variation of a magnesium chelatase subunit enhances photosynthesis influencing grain weight in wheat.
- Zhang et al. (2025) identified a mechanism based on proteolysis of the auxin transporter GhPIN3a that mediates regulation of cotton fiber development by light.
- Zhao et al. (2024) characterized a mutation in the transcription factor *GhMYB25-like* that affects fiber development and lint content in cotton.

## Data Availability

No new data were generated or analysed in support of this research.

## Dive Curated Terms

The following phenotypic, genotypic, and functional terms are of significance to the work described in this paper:
